# Supplementary material for: Biological Control Strategies and Integrated Arthropod Pest Management for Camellia oleifera
Source: Insects. 2025 Dec 9;16(12):1244. doi: 10.3390/insects16121244 (PMC12734399; doi:10.3390/insects16121244)
Supplement: Supplementary file 1 [file insects-16-01244-s001.zip › insects-4001230-supplementary.pdf]

**Supplementary Materials:** The following supporting information can be downloaded at: www.mdpi.com/xxx/s1,

**Table S1** List of viruses of oil-tea insect pests recorded in China

| Virus family | Insect host | Abbr. of virus name | Virus family | Insect host | Abbr. of virus name |
|---|---|---|---|---|---|
| **Baculoviridae** | | | | | |
| ***Nucleopolyhedrovirus*** | **Geometridae** | | | **Bombycidae** | |
| (14 species) | *Scopula subpunctaria* | SsNPV | | *Andraca bipunctata* | AbGV |
| | **Lymantridae** | | | **Eucleidae** | |
| | *Arctornis alba* Bremer | AaNPV | | *Cnidocampa flavescens* | CfGV |
| | **Eucleidae** | | | *Parasa bicolor* | PbGV |
| | *Clania variegata* | CvNPV | | *P. consocia* | PcGV |
| | *Mahasena colona* | McNPV | | *P. lepida* | PlGV |
| | *Chalcocelis albiguttata* | CaNPV | | *P. sinica* | PsGV |
| | *Cnidocampa flavescens* | CfNPV | | *Setora suberacta* | SsGV |
| | *Hyphorma minax* | HmNPV | | *T. sinensis* | TsGV |
| | *P. lepida* | PlNPV | | **Tortricidae** | |
| | *P. sinica* | PsNPV | | *Homona magnanima* | HmGV |
| | *Setora suberacta* | SsNPV | | *Adoxophyes pryvatana* | ApGV |
| | *Thosea baibarana* | TbNPV | ***Cypovirus*** | **Eucleidae** | |
| | *T. sinensis* | TsNPV | (7 species) | *Cnidocampa flavescens* | CfCPV |
| | **Noctuidae** | | | *Setora suberacta* | SsCPV |
| | *Agrotis ypsilon* | AyNPV | | *Thosea postornata* | TpCPV |
| | *Spodoptera litura* | SlNPV | | *T. sinensis* | TsCPV |
| ***Granulovirus*** | **Psychidae** | | | **Tortricidae** | |
| (15 species) | *Cryptothelea variegata* | CvGV | | *Homona magnanima* | HmCPV |
| | **Zygaenidae** | | | **Noctuidae** | |
| | *Eterusia aedea* | EaGV | | *Agrotis segetum* | AsCPV |
| | **Noctuidae** | | | *Agrotis ypsilon* | AyCPV |
| | *Spodoptera litura* | SlGV | ***Picornavirus*** | **Termitidae** | |
| | *Agrotis segetum* | AsGV | (1 species) | *Odontotemes formosanus* | OfPV |
| | *A. tokionis* | AtGV | | | |

**Table S2** List of entomophageous fungi from oil-tea pests in China

| Species | Host insect | Species | Host insect |
|---|---|---|---|
| **Zygomycotina** | | **Basidiomycotina** | |
| **Zygomycetes** | | **Pucciniomycetes** | |
| *Entomophthora aphidis* | *Toxoptera aurantii* | *Septobasidium pedicellatum* | Several species of scales |
| *Entomophthora fresenii* | *Toxoptera aurantii* | **Deuteromycotina** | |
| *Eryia radicans* | *Empoasca vitis* | **Coelomycetes** | |
| *Pandora borea* | *Toxoptera aurantii* | *Aschersonia aleyrodis* | *Aleurocanthus spiniferus, Dialeurodes citris* |
| **Ascomycota** | | *A. placenta* | *Aleurocanthus spiniferus, Dialeurodes citris* |
| **Sordariomycetes** | | **Hyphomycetes** | |
| *Cordyceps pruinosa* | *Iragoides fasciata, Thosea haibarana* | *Acremonium* sp. | *Aleurocanthus spiniferus* |
| *Cordyceps militaris* | *Iragoides fasciata, Thosea haibarana* | *Aspergillus oryzae* | *Aleurocanthus spiniferus* |
| *Hypocrella pulmae* | Ceroplastes ceriferus | *Aegerita webberi* | *Aleurocanthus spiniferus, Dialeurodes citri,* Several species of scales |
| *Leptobacillium muralicola* | Discovered in the habitat of oil-tea, no host has been identified yet. | *Beauveria bassiana* | *Empoasca vitis, Myllocerinus aurolineatus, Curculio chinensis,* Several species of moth |
| *Metapochonia bulbillosa* | Discovered in the habitat of oil-tea, no host has been identified yet. | *B. brongniartii* | *Adoxophyes orana orana* |
| *Metarhizium anisopliae* | *Empoasca vitis, Curculio chinensis, Basilepta melanopus* | *B. pseudobassiana* | *Empoasca vitis, Curculio chinensis* |
| *Metarhizium* Genus (*M. flavoviride, M. guizhouense, M. pemphigi, M. pinghaense, M. purpureogenum, M. robertsii, M. rileyi*) | Discovered in the habitat of oil-tea, no host has been identified yet. | *Cephalosporium coccorum* | *Aleurocanthus spiniferus, Ceroplastes ceriferus, Iragoides fasciata* |
| *Purpureocillium lilacinum* | Several species of aphids | *Cladosporium* sp. | *Aleurocanthus spiniferus* |
| *Isaria* Genus (*I. cateniannulata, I. tenuipes, I. farinosa, I. cateniobliqua*) | Discovered in the habitat of oil-tea, no host has been identified yet. | *Fusarium epuiseti* | *Aleurocanthus spiniferus* |
| **Eurotiomycetes** | | *Paecilomyces* sp. | *Aleurocanthus spiniferus* |
| *Hirsutella* sp. | *Aleurocanthus spiniferus* | *P. farinusus* | *Iragoides fasciata, Thosea haibarana* |
| *Mariannaea pruinasa* | *Iragoides fasciata, Thosea haibarana* | *P. fumoso-roseus* | *Iragoides fasciata* |
| | | *Pertaloeiopsis* sp. | *Aleurocanthus spiniferus* |
| | | *Pleurodesmostora coccorum* | *Aleurocanthus spiniferus* |
| | | *Trichothecium roseum* | *Aleurocanthus spiniferus* |

**Table S3** List of parasitic wasps associated with some common and serious oil-tea insect pests

| Host insects/development stage | Parasitic wasp species | | |
|---|---|---|---|
| **Lepidoptera** | | | |
| ***Clania minuscula*** | | ***Parametriotes theae*** | |
| Larval stage | **Ichneumonidae** | Laval stage | **Eucharitidae** |
| | *Coccygomimus disparis* | | *Goniozus japonicus* |
| | *C. luctuosus* | | **Bethyloidae** |
| | *C. nipponicus* | | *Goniozus sp.* |
| | *Echthromorpha agrestoria notulatoria* | | **Ichneumonidae** |
| | *Goryphus basilaris* | | *Temezucha philippinensis* |
| | *G. hyalinoides* | | **Braconidae** |
| | *Gregopimpla himalayensis* | | *Apanteles ruficrus* |
| | *Sericopimpla sagrae sauteri* | | *Apanteles schoenobii* |
| | *Temelucha philippinensis* | | *Apanteles theivorae* |
| | *Theronia atalantae gestator* | | *Hypoteromatus apantelophagus* |
| | *T. zebra diluta* | **Hemipotera** | |
| | *Xanthopimpla modesta modesta* | ***Empoasca vitis*** | |
| Pupal stage | **Chalcididae** | Egg stage | **Mymaridae** |
| | *Brachymeria lasus* | | *Anagrus* sp. |
| ***Adoxophyes orana orana*** | | Nymph and adult stage | **Dryinidae** |
| Egg stage | **Trichogrammatidae** | | *Aphelopus sinicus* |
| | *Trichogramma confusum* | | *A. theabius* |
| | *T. dendrolimi* | | *A. yanhuagi* |
| | **Braconidae** | ***Aleurocanthus spiniferus*** | |
| | *Apanteles conspersae* | Nymph and pupal stage | **Aphelinidae** |
| | *Chelonus pectinophorae* | | *Encarsia ishii* |
| | **Tetrastichidae** | | *E. longifasciata* |
| | *Tetrastichus* sp. | | *E. nipponica* |
| Pupal stage | **Chalcididae** | | *E. obtusiclava* |
| | *B. lasus* | | *E. opulenta* |
| ***Ectropis obliqua*** | | | *E. smithi* |
| Egg stage | **Trichogrammatidae** | | *E. serius* |

| | | | |
|---|---|---|---|
| | *Trichogramma confusum* | | *E. silvestrii* |
| | **Braconidae** | | **Platygasteridae** |
| | *Apanteles* sp1 | | *Amitus hesperidum* |
| | *Apantelse* sp2 | | *A. longicornis* |
| ***Buzura supressaria*** | | ***Ceroplastes ceriferus*** | |
| Egg stage | **Scelionidae** | Nymph stage | **Encyrtidae** |
| | *Telenomus buzurae* | | *Anicetus annulatus* |
| Larval stage | **Ichneumonidae** | | *A. benificus* |
| | *Cratichneumon* sp. | | *A. ceroplastis* |
| | *Therion* sp. | | *A. ohgushii* |
| | **Eulophidae** | | *Microterys clauseni* |
| | *Euplectrus* sp. | | *M. nietneri* |
| ***Euproctis pseudoconspersa*** | | | *M. speciosus* |
| Egg stage | **Trichogrammatidae** | | **Aphelinidae** |
| | *Trichogramma confusum* | | *Coccophagus hawaiiensis* |
| | *T. dendrolimi* | | *C. japonicus* |
| Larval stage | **Ichneumonidae** | | *C. scutellaris* |
| | *Xanthopimpla brachyparea* | ***Toxoptera aurantii*** | |
| | **Braconidae** | Nymph stage | **Aphidiidae** |
| | *A. conspersae* | | *Ephedrus persicae* |
| | *Meteorus japonicuis* | | *Ephedrus plagiator* |
| | **Chalcididae** | | *Ephedrus persicae* |
| | *Brachymeria obscurata* | | *Lipolexis gracilis* |
| ***Casmara patrona*** | | | *Lysiphlebus japonicus* |
| Laval stage | **Ichneumonidae** | | *Trixys sinensis* |
| | *Eriborus teretbrans* | | *Trixys toxopterae* |
| | *Gasteruption sp.* | | **Aphelinidae** |
| | **Braconidae** | | *Aphelinus mali* |
| | *Apanteles sp.* | | |
| | *Macrocentrus linearis* | | |
| | *Macrocentrus homonae* | | |
| | *Myosoma yanoi* | | |

**Table S4** Prominent vertebrate predators of oil-tea pests in China

| Class | Family | Species | Common name | Major preys |
|---|---|---|---|---|
| **Amphibia** | Bufonidae | *Bufo gargarizans* | Asiatic toad | *Scopula subpunctaria* |
| | Ranidae | *Rana limnocharis* | Cricket frog | *S. subpunctaria* |
| | | *R. nigromaculata* | Black dot frog | Tortricidae |
| **Aves** | Paridae | *Parus major* | Great tit | *Arctornis alba, Eterusia aedea, Toxoptera aurantii,* Psychidae, Limacodidae |
| | Laniidae | *Lanius cristatus* | Brown shrike | *Clania variegata* |
| | | *L. schach* | Long-tailed shrike | *Andraca bipunctata, Agrotis ypsilon, Anomala corpulenta,* |
| | | *L. tigrinus* | Tiger shrike | *C. variegata, Myllocerinus aurolineatus* |
| | Hirundinidae | *Hirundo rustica* | Barn swallow | *S. subpunctaria* |
| | | *H. daurica* | Red-rumped swallow | *M. aurolineatus* |
| | Pycnonotidae | *Pycnonotus sinensis* | Chinese bulbul | *M. aurolineatus* |
| | Muscicapidae | *Paradoxornis webbianus* | Vinous-throated parrotbill | *T. aurantii* |
| | Corridae | *Cyanopica cvana* | Azure-winged magpie | *Iragoides fasciata, Eumeta minuscula, C. variegata* |
| | | *Pica pica* | Magpie | *M. aurolineatus, C. variegata* |
| | Motacillidae | *Dendronanthus indicus* (Gmelin) | Forest wagtail | *S. subpunctaria, Thosea sinensis, Latoia sinica* |
| | | *Anthus hodysoni* | Olive-backed pipit | *M. aurolineatus* |
| **Mammalia** | Muridae | *Leopoldamys edwards* | Edward's Rat | *Curculio chinensis* |

**Table S5** List of spiders and predatory mites recorded in oil-tea gardens in Hunan, China

| | | | | | |
|---|---|---|---|---|---|
| Araneae | Thomisidae | *Misumenops tricuspidatus* | Araneae | Salticidae | *Icius linea* |
| | | *Oxytate parappela* | | | *Jotus diffioilis* |
| | | *Pholodromus spinitarsis* | | | *Jotus munitus* |
| | | *Pholodromus cespitum* | | | *Marpissa magister* |
| | | *Phololromus sulaureolus* | | | *Marpissa elongata* |
| | | *Runcimia albostriata* | | | *Plerippus setipes* |
| | | *Synaema globosum* | | | *Plexippus* sp. |
| | | *Thomisus labetectus* | | | *Menemerus confusua* |
| | | *Thanatus farmicinus* | | | *Menemerus hvmeshimensis* |
| | | *Tibellus tenellus* | | | *Evarcha alaria* |
| | | *Tibillus* sp. | | | *Myrmarachme japonmica* |
| | | *Tmarus orientalis* | | | *Myrmarachme gisti* |
| | | *Xysticus ephippiatus* | | | *Sitticus sinensis* |
| | Araneidae | *Argiope bruennichi* | | Oxyopidae | *Oxyopes sertatus* |
| | | *Araneus ventriclisus* | | | *Oxyopes macilentus* |
| | | *Araneus sia* | | | *Oxyopes daksina* |
| | | *Araneus ejusmodi* | | | *Poecilochroa hvsiziro* |
| | | *Araneus alternidens* | | Theridiidae | *Theridion pinastri* |
| | | *Araneus cornutus* | | | *Theridion angulithorax* |
| | | *Araneus dispicatus* | | | *Theridion octomacaclatum* |
| | | *Araneus ishisalvai* | | | *Theridion tepidaiioum* |
| | | *Araneus variegatus* | | | *Theridion latifutium* |
| | | *Argiope amoena* | | Micryphantidae | *Erigonidium graminicolum* |
| | | *Argiope lobata* | | Lycosidae | *Lycosa sinensis* |
| | | *Cyclosa ginnaga* | | | *Lycosa pseudoannulata* |
| | | *Cyclosa ginnaga* | | | *Lycosa coelescris* |
| | | *Gusterucantha kuhlii* | | | *Pardosa astrigera* |
| | | *Larinia angiopifirmis* | | | *Pirata subpiracicus* |

| | | | | | |
|---|---|---|---|---|---|
| | | *Neoscona doenitzi* | | Ganphosidae | *Gnaphosa kompirensis* |
| | | *Neoscona theisi* | | Platoridae | *Plator ripporicus* |
| | | *Neoscona fuscocolorata* | | Clubionidae | *Clubiona japonicola* |
| | | *Nephila clavata* | | | *Clubiona werulescors* |
| | | *Singa pygmaea* | | | *Clubiona hummedi* |
| | | *Singa hamata* | | | *Chiracanthium virescens* |
| | Tetragnathidae | *Leucauge blauda* | | | *Chiracanthium pennyi* |
| | | *Leucauge decorata* | | Hreropodidae | *Heteropoda licenti* |
| | | *Tetragnatha shikokiana* | | Dictynidae | *Dictyna arundinacea* |
| | | *Tetragnatha maxillosa* | | Segestrudae | *Aarisdna lateralis* |
| | | *Tetragnatha cliens* | | Sicarudae | *Scytodes thoracica* |
| | | *Tetragnatha squamata* | | Pholcidae | *Pholcus* sp. |
| | Salticidae | *Bianor aenescens* | | Urocteidae | *Urocfea lesserti* |
| | | *Cawbotus atratus* | | Agelenidae | *Agelena labyrinthica* |
| | | *Euophrys undulatouvittata* | | | *Agelena difficilis* |
| Mesostigmata | Phytoseiidae | *Amblyseius deleoni* | | Atypidae | *Atypus sinensis* |
| | | *Amblyseius hericolus* | | Eresidae | *Eresus niger* |
| | | *Amblyseius nicholsi* | | | |
| | | *Typhlodromus* sp. | | | |
| | Stigmaeidae | *Agistemus exsertus* | | | |
| | Anystidae | *Anystis* sp. | | | |
| | Thrombidiidae | *Allothrombium* sp. | | | |
